# Supplementary material for: Lyme disease and relapsing fever in Mexico: An overview of human and wildlife infections
Source: PLoS One. 2020 Sep 17;15(9):e0238496. doi: 10.1371/journal.pone.0238496 (PMC7497999; doi:10.1371/journal.pone.0238496)
Supplement: S1 Table — (DOCX) [file pone.0238496.s001.docx]

| *BORRELIA* | | | HUMAN CASES | | | VECTORS | DETECTION METHOD | LOCALITY | REFERENCE |
| --- | --- | --- | --- | --- | --- | --- | --- | --- | --- |
| Group | **Species** | **No. of cases** | **Sex** | **Group of age** | **Community type** | **Family** | **Clinical manifestation/diagnostic test** | **State** |  |
| RF | *Borrelia* sp. (relapsing fever) | 2 | Male | ND | Rural | ND | Thick drop | Aguascalientes | [1] |
|  |  | 1 | Female | ND | Rural | ND | Thick drop | Aguascalientes | [1] |
|  |  | 1 | Female | Adult | Urban | ND | Wright's stain | Sonora | [2] |
|  |  | 1 | Female | Adult | Rural | ND | Western Blot (WB) | Sonora | [3] |
| BL | *Borrelia afzelii* | 2 | ND | ND | Urban | ND | Enzyme-Linked Immunosorbent Assay (ELISA)/WB | Mexico City | [4] |
|  | *Borrelia burgdorferi* s.l. | 1 | Female | Adult | Urban | ND | WB | Mexico City | [5] |
|  |  | 2 | Female | Adult | Urban | ND | ELISA/WB | Nuevo Leon | [6] |
|  |  | 2 | Female | Adult | Urban | ND | ELISA/WB | Nuevo Leon | [6] |
|  |  | 1 | Female | Child | Urban | ND | ELISA/WB | Coahuila | [7] |
|  |  | 1 | Female | Child | Urban | ND | General clinical manifestations | Mexico City | [8] |
|  |  | 1 | Female | Child | Urban | ND | ELISA | Nuevo Leon | [9] |
|  |  | 4 | Female | ND | ND | ND | ELISA/WB | Nuevo Leon | [10] |
|  |  | 21 | Female | ND | Urban | ND | *Erythema migrans,* arthritis/ELISA | Sinaloa | [11] |
|  |  | 1 | Female | ND | Rural | ND | Enzyme-Linked Fluorescent Assay (ELFA) | Yucatán | [12] |
|  |  | 97 | Female | ND | ND | ND | ELISA/WB | ND | [13] |
|  |  | 3 | Male | Adult | Urban | ND | ELISA/WB | Nuevo Leon | [6] |
|  |  | 1 | Male | Adult | Urban | ND | ELISA/WB | Nuevo Leon | [6] |
|  |  | 1 | Male | Adult | Urban | ND | ELISA/WB | Nuevo Leon | [6] |
|  |  | 1 | Male | Adult | Urban | ND | WB | ND | [14] |
|  |  | 1 | Male | Adult | Rural | Ixodidae | WB | Morelos | [15] |
|  |  | 1 | Male | Child | Urban | ND | ELISA | Nuevo Leon | [9] |
|  |  | 5 | Male | ND | ND | ND | ELISA/WB | Nuevo Leon | [10] |
|  |  | 11 | Male | ND | Urban | ND | *Erythema migrans,* arthritis/ELISA | Sinaloa | [11] |
|  |  | 71 | Male | ND | ND | ND | ELISA/WB | ND | [13] |
|  |  | 1 | ND | ND | ND | ND | ELISA/WB | Coahuila | [16] |
|  |  | 20 | ND | Child | Urban | ND | ELISA/WB/PCR | Estado de México, Morelos, Hidalgo, Tabasco | [17] |
|  |  | 1 | ND | ND | ND | ND | ELISA/WB | Mexico City | [16] |
|  |  | 1 | ND | ND | ND | ND | ELISA/WB | Nuevo Leon | [16] |
|  |  | 1 | ND | ND | ND | ND | ELISA/WB | Tamaulipas | [16] |
|  |  | 1 | ND | ND | ND | ND | ELISA/WB | Veracruz | [16] |
|  |  | 2 | ND | ND | ND | ND | PCR | ND | [18] |
|  | *Borrelia burgdorferi* s.s. | 1 | ND | Adult | Rural | ND | ELISA/WB | Estado de México | [19] |
|  |  | 1 | ND | Adult | Rural | Ixodidae | ELISA/WB | Estado de México | [19] |
|  |  | 1 | Male | Adult | Rural | ND | ELISA/WB/PCR | Veracruz | This study |
|  |  | 1 | ND | Adult | ND | Ixodidae | PCR | Quintana Roo | [19] |
|  |  | 1 | ND | Child | Rural | ND | ELISA/WB | Estado de México | [19] |
|  |  | 7 | ND | ND | Urban | ND | WB | Coahuila | [20] |
|  |  | 25 | ND | ND | Urban | ND | WB | Mexico City | [20] |
|  |  | 2 | ND | ND | Urban | ND | ELISA/WB | Mexico City | [4] |
|  |  | 47 | ND | ND | Urban | ND | WB | Nuevo Leon | [20] |
|  |  | 43 | ND | ND | Urban | ND | WB | Tamaulipas | [20] |
|  | *Borrelia garinii* | 10 | ND | ND | Urban | ND | ELISA/WB | Mexico City | [4] |

**REFERENCES**

1. Pilz H, Mooser H. La Fiebre Recurrente en Aguascalientes. Boletín del Inst Hig. 1936;1: 295–300.

2. Sotelo-Cruz N, Valencia-Mayoral P. Borreliosis, fiebre recurrente causada por espiroquetas. Informe de un caso caso. Bol Med Hosp Infant Mex. 2012;69: 121–125.

3. Vázquez-Guerrero E, Adan-Bante NP, Mercado-Uribe MC, Hernández-Rodríguez C, Villa-Tanaca L, Lopez JE, et al. Case report: A retrospective serological analysis indicating human exposure to tick-borne relapsing fever spirochetes in Sonora, Mexico. PLoS Negl Trop Dis. 2019;13: e0007215. doi:10.1371/journal.pntd.0007215

4. Gordillo-Pérez G, García-Juárez I, Solórzano-Santos F, Corrales-Zúñiga L, Muñoz-Hernández O, Torres-López J. Serological evidence of *Borrelia* *burgdorferi* infection in mexican patients with facial palsy. Rev Investig Clin. 2017;69: 344–348. doi:10.24875/RIC17002344

5. Guevara-Valmaña OI, Martínez-Jiménez A, Mendoza-García JG. Lyme disease in Mexico City. Med Interna Mex. 2019;35: 435–440. doi:10.24245/mim.v35i3.2276

6. Skinner-Taylor CM, Flores MS, Salinas JA, Arevalo-Niño K, Galán-Wong LJ, Maldonado G, et al. Antibody profile to *Borrelia* *burgdorferi* in veterinarians from Nuevo León, Mexico, a non-endemic area of this zoonosis. Reumatologia. 2016;54: 97–102. doi:10.5114/reum.2016.61208

7. Rodríguez-García A, Arroyo-Garza I, Patiño-Ramirez BE. Uveitis associated with Lyme disease in Mexico. Rev Mex Oftalmol (English Ed. 2020;94. doi:10.24875/rmoe.m20000093

8. Vargas MH. Enfermedad de Lyme en la CIudad de México. Salud Publica Mex. 1993;35: 435–436.

9. Arroyave CM, Tamez-González R. Enfermedad de Lyme. Informe de dos casos. Bol Med Hosp Infant Mex. 1994;51: 117–121.

10. Skinner-Taylor CM, Flores-González MS, Esquivel-Valerio JA, Salinas-Meléndez JA, Salinas-Palacios CK, Rodríguez-Amado J, et al. Evidencia de la enfermedad de Lyme en una población de alto riesgo del noreste de México. Med Universiitaria. 2007;9: 105–111. Available: http://new.medigraphic.com/cgi-bin/resumenMain.cgi?IDARTICULO=18522

11. Maradiaga-Ceceña MA, Llausas-Vargas A, Baquera-Heredia J, Kúmate-Rodríguez J. Eritema crónico migratorio asociado a artritis. Enfermedad de Lyme o una variante. Rev Mex Reumatol. 1991;6: 61.

12. Macari-Jorge A, Cárdenas-Marrufo MF, Peniche-Lara G. Seroprevalencia de infección por *Borrelia* *burgdorferi* en una población rural ocupacionalmente expuesta de Yucatán, México. Rev Latinoam Patol Clínica y Med Lab. 2017;64: 4–7.

13. Gordillo-Pérez G, Solorzano F, Cervantes-Castillo A, Sanchez-Vaca G, García-Ramirez R, Díaz AM, et al. Lyme Neuroborreliosis is a Severe and Frequent Neurological Disease in Mexico. Arch Med Res. 2018;49: 399–404. doi:10.1016/j.arcmed.2018.11.007

14. Garcia-Toribio MG. Description of neurophysiological findings in mononeuritis multiple as a neurologic manifestation of *Borrelia* *burgdorferi* infection - Case report. Clin Neurophysiol. 2018;129: e87–e88. doi:10.1016/j.clinph.2018.04.219

15. García-Frade-Ruiz LF. Enfermedad de Lyme adquirida en el estado de Morelos, México. Med Interna México. 2018;34: 342–348. doi:10.24245/mim.v34i2.1605

16. Gordillo G, Torres J, Solorzano F, Cedillo-Rivera R, Tapia-Conyer R, Muñoz O. Serologic evidences suggesting the presence of *Borrelia* *burgdorferi* infection in Mexico. Arch Med Res. 1999;30: 64–68. doi:10.1016/S0188-0128(98)00015-3

17. Gordillo-Pérez G, Solórzano-Santos F. Enfermedad de Lyme. Experiencia en niños mexicanos. Bol Med Hosp Infant Mex. 2010;67: 164–176.

18. Salinas-Meléndez JA, Tamez-González R, Welsh-Lozano O, Barrera-Saldaña HA. Detection of *Borrelia* *burgdorferi* DNA in human skin biopsies and dog synovial fluid by the polymerase chain reaction. Rev Latinoam Microbiol. 1995;37: 7–10.

19. Gordillo-Pérez G, Torres J, Solórzano-Santos F, De Martino S, Lipsker D, Velázquez E, et al. *Borrelia* *burgdorferi* infection and cutaneous Lyme disease, Mexico. Emerg Infect Dis. 2007;13: 1556–1558. doi:10.3201/eid1310.060630

20. Gordillo-Pérez G, Torres J, Solórzano-Santos F, Garduño-Bautista V, Tapia-Conyer R, Muñoz O, et al. Estudio seroepidemiológico de borreliosis de Lyme en la Ciudad de México y el noreste de la República Mexicana. Enferm Infecc Microbiol Clin. 2003;11: 480–489. doi:10.1590/S0036-36342003000500004
