# Supplementary material for: Lyme disease and relapsing fever in Mexico: An overview of human and wildlife infections
Source: PLoS One. 2020 Sep 17;15(9):e0238496. doi: 10.1371/journal.pone.0238496 (PMC7497999; doi:10.1371/journal.pone.0238496)
Supplement: S2 Table — (DOCX) [file pone.0238496.s002.docx]

| *BORRELIA* | | | MAMMALIAN HOST | | VECTOR | DETECTION METHOD | LOCALITY | REFERENCE |
| --- | --- | --- | --- | --- | --- | --- | --- | --- |
| Group | **Species** | **No. of reports** | **Order** | **Species** | **Species** |  | **State** |  |
| RF | *Borrelia duguesii* | 1 | Rodentia | *Neotoma micropus* | *Ornithodoros duguesi* | Natural transmition | Coahuila | [1] |
|  | *Borrelia mazzottii* | 1 | ND | ND | *Ornithodoros talaje* | Natural transmition | ND | [2] |
|  | *Borrelia turicatae* | 406 | ND | ND | *Ornithodoros turicata* | Natural transmition | Aguascalientes | [3] |
|  |  | 142 | ND | ND | *Ornithodoros turicata* | Natural transmition | Guanajuato | [3] |
|  |  | 1 | ND | ND | *Ornithodoros turicata* | Natural transmition | San Luis Potosi | [3] |
| BL | *Borrelia burgdorferi* s.l. | 2 | Artiodactyla | *Odocoileus virginianus* | ND | Enzyme-Linked Immunosorbent Assay (ELISA)/Western Blot (WB) | Coahuila | [4] |
|  |  | 3 | Artiodactyla | *Odocoileus virginianus* | ND | ELISA/WB | Nuevo Leon | [4] |
|  |  | 2 | Artiodactyla | *Odocoileus virginianus* | ND | ELISA/WB | ND | [4] |
|  |  | 14 | Carnivora | *Canis lupus familiaris* | ND | ELISA | Baja California | [5] |
|  |  | 46 | Carnivora | *Canis lupus familiaris* | ND | ELISA | Baja California | [6] |
|  |  | 136 | Carnivora | *Canis lupus familiaris* | ND | Immunofluorescence assay (IFA) | Nuevo Leon | [7] |
|  |  | 4 | Carnivora | *Canis lupus familiaris* | ND | ELISA | Nuevo Leon | [7] |
|  |  | 1 | Carnivora | *Canis lupus familiaris* | ND | PCR | Nuevo Leon | [8] |
|  |  | 1 | Carnivora | *Canis lupus familiaris* | ND | PCR | Nuevo Leon | [8] |
|  |  | 1 | Carnivora | *Panthera onca* | *Ixodes scapularis* | PCR | Tamaulipas | [9] |
|  |  | 5 | Carnivora | *Canis lupus familiaris* | *Rhipicephalus sanguineus* | PCR | Yucatán | [10] |
|  |  | 8 | Carnivora | *Canis lupus familiaris* | *Rhipicephalus sanguineus* | PCR | Yucatán | [10] |
|  |  | 5 | Carnivora | *Canis lupus familiaris* | *Amblyomma mixtum* | PCR | Yucatán | [10] |
|  |  | 5 | Carnivora | *Canis lupus familiaris* | *Ixodes affinis* | PCR | Yucatán | [10] |
|  |  | 8 | Carnivora | *Canis lupus familiaris* | *Amblyomma mixtum* | PCR | Yucatán | [10] |
|  |  | 8 | Carnivora | *Canis lupus familiaris* | *Ixodes affinis* | PCR | Yucatán | [10] |
|  |  | 13 | Carnivora | *Canis lupus familiaris* | ND | PCR | Yucatán | [10] |
|  |  | 12 | Carnivora | *Canis lupus familiaris* | ND | PCR | Yucatán | [10] |
|  |  | 1 | Carnivora | *Canis lupus familiaris* | ND | PCR | ND | [11] |
|  |  | 4 | Carnivora | *Canis lupus familiaris* | ND | ELISA | ND | [12] |
|  |  | 8 | Lagomorpha | *Sylvilagus floridianus* | *Ixodes scapularis* | PCR | Nuevo Leon | [9] |
|  |  | 1 | Perissodactyla | *Equus caballus* | ND | IFA/WB | Nuevo Leon | [13] |
|  |  | 1 | Rodentia | *Heteromys pictus* | *Ixodes scapularis* | PCR | Nuevo Leon | [9] |
|  |  | 2 | Rodentia | *Microtus mexicanus* | ND | PCR | Michoacan | [14] |
|  |  | 20 | Rodentia | *Mus musculus* | ND | PCR | Yucatan | [15] |
|  |  | 20 | Rodentia | *Mus musculus* | ND | PCR | Yucatan | [15] |
|  |  | 3 | Rodentia | *Neotomodon alstoni* | ND | PCR | Mexico | [14] |
|  |  | 3 | Rodentia | *Neotomodon alstoni* | ND | PCR | Mexico | [14] |
|  |  | 2 | Rodentia | *Neotoma mexicana* | ND | PCR | Michoacan | [14] |
|  |  | 6 | Rodentia | *Peromyscus maniculatus* | ND | PCR | Mexico | [14] |
|  |  | 1 | Rodentia | *Peromyscus maniculatus* | ND | PCR | Mexico | [14] |
|  |  | 3 | Rodentia | *Peromyscus leucopus* | ND | PCR | Mexico | [14] |
|  |  | 2 | Rodentia | *Rattus rattus* | ND | PCR | Yucatan | [15] |
|  |  | 3 | Rodentia | *Rattus rattus* | ND | PCR | Yucatan | [15] |
|  |  | 2 | ND | ND | *Dermacentor variabilis* | PCR | Nuevo Leon | [8] |
|  |  | 1 | ND | ND | *Dermacentor variabilis* | PCR | Nuevo Leon | [8] |
|  |  | 1 | ND | ND | *Dermacentor variabilis* | PCR | Nuevo Leon | [8] |
|  |  | 1 | ND | ND | *Ixodes spinipalpis* | PCR | ND | [14] |
|  |  | 1 | ND | ND | *Ixodes tovari* | PCR | ND | [14] |
|  |  | 2 | ND | ND | *Ixodes scapularis* | PCR | Tamaulipas | [9] |
|  | *Borrelia burgdorferi* s.s. | 2 | Artiodactyla | *Bos taurus* | *Amblyomma cajennense* | IFA/PCR/Southern Blot (SB) | Tamaulipas | [9] |
|  |  | 5 | Carnivora | *Basariscus astutus* | *Ixodes texanus* | WB/PCR | Nuevo Leon | [16] |
|  |  | 1 | Carnivora | *Basariscus astutus* | *Ixodes texanus* | IFA/PCR/SB | Nuevo Leon | [17] |
|  |  | 3 | Carnivora | *Canis lupus familiaris* | *Amblyomma cajennense* | IFA/PCR/SB | Tamaulipas | [17] |
|  |  | 1 | Carnivora | *Canis lupus familiaris* | *Ixodes kingi* | PCR | Chihuahua | [18] |
|  |  | 5 | Lagomorpha | *Sylvilagus floridianus chapmani* | *Ixodes scapularis* | WB/PCR | Nuevo Leon | [16] |
|  |  | 7 | Lagomorpha | *Sylvilagus floridianus* | *Ixodes scapularis* | IFA/PCR/SB | Nuevo Leon | [17] |
|  |  | 1 | Primates | *Homo sapiens sapiens* | *Amblyomma americanum* | WB/PCR | Jalisco | [16] |
|  |  | 1 | ND | ND | *Amblyomma cajennense* | WB/PCR | Nuevo Leon | [16] |
|  |  | 2 | ND | ND | *Amblyomma cajennense* | WB/PCR | Tamaulipas | [16] |
|  |  | 1 | ND | ND | *Amblyomma cajennense* | WB/PCR | Tamaulipas | [16] |
|  |  | 6 | ND | ND | *Amblyomma cajennense* | IFA/PCR/SB | Nuevo Leon | [17] |
|  |  | 1 | ND | ND | *Dermacentor andersoni* | IFA/PCR/SB | Tamaulipas | [17] |

**REFERENCES**

1. Mazzotti L. Sobre una nueva espiroqueta de la fiebre recurrente, encontrada en México.pdf. Rev Inst Salubr Enferm Trop. 1949;10: 277–281.

2. Davis GE. A relapsing fever spirochete, *Borrelia* *mazzottii* (sp. nov.), from *Ornithodoros* *talaje* from Mexico. Am J Epidemiol. 1956;63: 13–17. doi:10.1093/oxfordjournals.aje.a119787

3. Brumpt E, Mazzotti L, Brumpt LC. Étude épidémiologique de la fièvre récurrente endémique des hauts plateaux Mexicains. Ann Parasitol Hum Comparée. 1939;17: 275–286. doi:10.1051/parasite/1939-1940174275

4. Martinez A, Salinas A, Martinez F, Cantu A, Miller DK. Serosurvey for selected disease agents in white-tailed deer from Mexico. J Wildl Dis. 1999;35: 799–803. doi:10.7589/0090-3558-35.4.799

5. Tinoco-Gracia L, Quiroz-Romero H, Quintero-Martínez MT, Rentería-Evangelista TB, Barreras-Serrano A, López-Valencia G, et al. Seroprevalence of *Borrelia* *burgdorferi* in dogs froma a Mexico-US border desert region: Pilot study. Journal of Animal and Veterinary Advances. 2007. pp. 787–789.

6. Tinoco-Gracia L, Quiroz-Romero H, Quintero-Martínez MT, Rentería-Evangelista TB, Barreras-Serrano A, Hori-Oshima S, et al. Prevalence and risk factors for *Borrelia* *burgdorferi* infection in dogs of animal control centers from Mexicali, Baja California: A Mexico-US Border City. J Anim Vet Adv. 2009;8: 251–254.

7. Salinas-Melendez JA, Zarate-Ramos JJ, Avalos-Ramirez R, Hernandez-Escareno JJ, Guzman-Acosta G, Riojas-Valdes VM, et al. Prevalence of antibodies against *Borrelia* *burgdorferi* in dogs from Monterrey, Mexico. J Anim Vet Adv. 2011;10: 2720–2723. doi:10.3923/javaa.2011.2720.2723

8. Galaviz-Silva L, Pérez-Treviño KC, Molina-Garza ZJ. Distribution of ixodid ticks on dogs in Nuevo León, Mexico, and their association with *Borrelia* *burgdorferi* sensu lato. Exp Appl Acarol. 2013;61: 491–501. doi:10.1007/s10493-013-9707-5

9. Feria-Arroyo TP, Castro-Arellano I, Gordillo-Perez G, Cavazos AL, Vargas-Sandoval M, Grover A, et al. Implications of climate change on the distribution of the tick vector *Ixodes* *scapularis* and risk for Lyme disease in the Texas-Mexico transboundary region. Parasites and Vectors. 2014;7: 199. doi:10.1186/1756-3305-7-199

10. Solís-Hernández A, Rodríguez-Vivas RI, Esteve-Gasent MD, Villegas-Pérez SL. Detección de *Borrelia* *burgdorferi* sensu lato en perros y sus garrapatas en comunidades rurales de Yucatán, México. Rev Biol Trop. 2018;66: 428–437.

11. Salinas-Meléndez JA, Tamez-González R, Welsh-Lozano O, Barrera-Saldaña HA. Detection of *Borrelia* *burgdorferi* DNA in human skin biopsies and dog synovial fluid by the polymerase chain reaction. Rev Latinoam Microbiol. 1995;37: 7–10.

12. Movilla R, García C, Siebert S, Roura X. Countrywide serological evaluation of canine prevalence for *Anaplasma* spp., *Borrelia* *burgdorferi* (sensu lato), *Dirofilaria* *immitis* and *Ehrlichia* *canis* in Mexico. Parasites and Vectors. Parasites & Vectors; 2016;9: e421. doi:10.1186/s13071-016-1686-z

13. Salinas-Meléndez JA, Galván de la Garza S, Riojas-Valdés VM, Wong González A, Ávalos-Ramírez R. Antibody detection against *Borrelia* *burgdorferi* in horses located in the suburban areas of Monterrey, Nuevo León. Rev Latinoam Microbiol. 2001;43: 161–164.

14. Gordillo-Pérez G, Vargas-Sandoval M, Sosa-Gutiérrez CG, Minero-González E, Schroeder-Lima E, Lara-Chávez BN, et al. Prevalencia de Infección de *Borrelia* *burgdorferi* y *Ehrlichia* spp. en garrapatas y roedores provenientes de tres parques nacionales del Centro de la República Mexicana. Acarol Latinoam. 2012;1: 291–295.

15. Solís-Hernández A, Rodríguez-Vivas RI, Esteve–Gassent MD, Villegas-Pérez SL. Prevalencia de *Borrelia* *burgdorferi* sensu lato en roedores sinantrópicos de dos comunidades rurales de Yucatán, México. Biomédica. 2016;36: 109–117. doi:10.7705/biomedica.v36i3.3139

16. Vargas M, Gordillo-Pérez G, Solórzano F, Rivera A, Polaco OJ, Muñoz O, et al. Evidences of *Borrelia* *burgdorferi* in ticks of the northeast of Mexico. In: Estrada-Venegas EG, Equihua-Martínez A, Luna-León C, Rosas-Acevedo JL, editors. Entomología mexicana. Sociedad Mexicana de Entomología; 2007. pp. 830–835.

17. Gordillo-Pérez G, Vargas M, Solórzano-Santos F, Rivera A, Polaco OJ, Alvarado L, et al. Demonstration of *Borrelia* *burgdorferi* sensu stricto infection in ticks from the northeast of Mexico. Clin Microbiol Infect. 2009;15: 496–498. doi:10.1111/j.1469-0691.2009.02776.x

18. López-Pérez AM, Sánchez-Montes S, Foley J, Guzmán-Cornejo C, Colunga-Salas P, Pascoe E, et al. Molecular evidence of *Borrelia* *burgdorferi* sensu stricto and *Rickettsia* *massiliae* in ticks collected from a domestic-wild carnivore interface in Chihuahua, Mexico. Ticks Tick Borne Dis. 2019;10. doi:10.1016/j.ttbdis.2019.05.018
