# Supplementary material for: Lyme disease and relapsing fever in Mexico: An overview of human and wildlife infections
Source: PLoS One. 2020 Sep 17;15(9):e0238496. doi: 10.1371/journal.pone.0238496 (PMC7497999; doi:10.1371/journal.pone.0238496)
Supplement: Attachment — Submitted filename: 20.docx [file pone.0238496.s003.docx]

General Comments

The manuscript by Colunga-Salas and colleagues aims to "compile all the information published on Borrelia in Mexico and to present actual epidemiological trends." Although this is a noble goal, the epidemiological data appears to be too sparse and fragmented to achieve this goal. The other feature of the report is the detailed genomic description of the first documented endemic case of Borrelia burgdorferi sensu stricto (Bbss) in Mexico. Reorganization of the manuscript to focus on this case would make sense.

Major Comments

1. The detailed genomic description of the first documented endemic case of Bbss in Mexico is certainly original and should be the major focus of the report. The manuscript should include the actual BLAST sequence from that case to confirm its validity. Starting with this case will show where future studies should go in determining the epidemiology of tick-borne disease in Mexico. In addition, a detailed travel history should be given because the patient may have acquired his infection outside of Mexico.

2. The epidemiology of Borrelia burgdorferi (Bb) and relapsing fever Borrelia (RFB) in Mexico is certainly of interest, but the available data was limited to 18 states, thereby introducing significant selection bias into the study results. Presentation of this review data could be done in a more organized and concise format with less speculation about the implications of the incomplete data.

3. A benefit of separating out the case report from the epidemiology is that the case can be presented in a more organized fashion, with clinical features, test methodology, results and conclusions. Right now the clinical and epidemiology aspects are mixed together, and it is a mess.

4. Page 4, line 84: "The competent vectors of this bacterial species are hard ticks of the Ixodes ricinus complex". Ixodes ricinus is mainly found in Europe, so better to say that Bb is transmitted by hard ixodid ticks. The authors should also mention soft argasid ticks that transmit RFB (Fesler et al, Healthcare 2020;8:97).

5. For future review, it would be helpful to separate the Tables from the text. Much easier to follow the bouncing ball that way.

6. Page 15, line 221: Isn't it surprising that Bb was reported mostly in urban communities? The number of recognized RFB cases (5) was too small to draw geographical conclusions.

7. Numerous spelling and grammatical errors need to be corrected. Examples (comments): prompts to include both diseases as possible causes of reports on “fever of unknow origin” (awkward sentence and sp), disperse (sp), incriminated (implicated), widely dispersed (just the opposite: not disseminated), a red skin lesion after visiting the family farm that lasted several weeks (the lesion, not the visit), sensibility (sensitivity), short time (short term), starting performing (?), BL(LB?), sensorial (sensory), sub-sampling (inadequate sampling)

Minor Comments

1. Page 22, line 331: REP and MAB sentence is hard to understand

2. Reference 41 is incomplete
