# Supplementary material for: Lyme disease and relapsing fever in Mexico: An overview of human and wildlife infections
Source: PLoS One. 2020 Sep 17;15(9):e0238496. doi: 10.1371/journal.pone.0238496 (PMC7497999; doi:10.1371/journal.pone.0238496)
Supplement: Attachment — Submitted filename: Response to Reviewers.docx [file pone.0238496.s004.docx]

**Response to Reviewers**

We thank the reviewers for their comments and have now addressed all their points making the pertinent changes throughout the manuscript. The detailed response are included below.

**REVIEWER #1**

***(1) The term "borreliosis" for both Lyme disease and relapsing fever is not common usage. This will likely confuse readers. Why not just write about Lyme disease (or Lyme borreliosis) and relapsing fever?***

**Response**: We agree with the reviewer and changed the term “borreliosis” in the abstract. Additionally, we proposed changes in the title and short title, and made changes throughout the manuscript (Lines 63, 108, 112, 243, 248, 363, 420, 470, 490).

***(2) The article is mainly a case report with a review of the literature. It is not a research article. Table 1 is more appropriate for a textbook chapter.***

**Response:** One of the aims of the study was to assess the epidemiological situation of borreliosis in Mexico, since most of the studies done so far are not easily available. An epidemiological update is needed to incentivize studies on Lyme borreliosis and Relapsing fever and to consider Relapsing fever as an important differential diagnosis in cases of “Fever of Unknown Origin” in Mexico.

Regarding Table 1, we consider it important to update knowledge on species that are pathogenic for both humans and animals, for readers not familiar with the genus *Borrelia*.

***(3) The nucleotide sequence with accession number of MN607028 (line 165) is not publicly available. This is a major problem.***

**Response:** We are aware of this situation, however, when submitting the sequence to GenBank, it was requested that it be released in 2022 or earlier, if the accession number was published. Despite this inconvenience, the publisher asked us for the sequence, which we shared. Since we don’t know whether this document was shared with the reviewer, we include the sequence:

>MexicanPatient

TTCCGATGCAGACAGAGGTTCTATACAAATTGAAATAGAGCAACTTACAGACGAAATTAATAGAATTGCTGATCAAGCTCAATATAACCAAATGCACATGTTATCAAACAAATCTGCTTCTCAAAATGTAAGAACAGCTGAAGAGCTTGGAATGCAGCCTGCAAAAATTAACACACCAGCATCACTTTCAGGGTCTCAAGCGTCTTGGACTTTAAGAGTTCATGTTGGAGCAAACCAAGATGAAGCTATTGCTGT

***(4) In any case, the examples of "B. burgdorferi" in the tree are not from a recognized reference strain, preferably the type strain for the species, but a short fragment of the flagellin gene from Illinois, a state with a low incidence of Lyme disease.***

**Response:** We thank the reviewer and have re-analyzed the set of sequences. Three B31 sequences were included (Figure 1). Accession numbers: AE000783 and CP019767.

Furthermore, changes were made in methods and figure legend 1, since new substitution model analyses was required, and a different substitution model was obtained from the new alignment (Lines 163-164, 168, 176).
 **REVIEWER #2**

***The manuscript is well written, and the authors were able to present their study well. The compilation of data presenting the epidemiological trend of Lyme Borreliosis and Tick borne Relapsing fever in Mexico will be helpful for further research in this field. However, I would suggest the authors proof read the manuscript for minor errors. For example: Relapsing Fever (RF) is written as FR in Table 1 under the Borrelia group column. Also, the full from of all abbreviations used in the manuscript needs to be given the first time it is mentioned in the paper.***

**Response**: We thank the reviewer and have carefully revised the abbreviations throughout the manuscript and made sure that the first time any abbreviation was used, it was fully written. Changes were done throughout the manuscript (Lines 162-163, 215-216, 237). We also corrected the error (changing FR to RF) in Table 1.

**REVIEWER #3**

***(1) General Comments
The manuscript by Colunga-Salas and colleagues aims to "compile all the information published on Borrelia in Mexico and to present actual epidemiological trends." Although this is a noble goal, the epidemiological data appears to be too sparse and fragmented to achieve this goal. The other feature of the report is the detailed genomic description of the first documented endemic case of Borrelia burgdorferi sensu stricto (Bbss) in Mexico. Reorganization of the manuscript to focus on this case would make sense.***

**Response:** We appreciate the recommendation and have now re-organized the manuscript into sections (A), related to the case report and section (B) related to review of the literature. The case report is now separated and fully presented after the introduction (Lines 114-180).

Regarding the epidemiological panorama, we are aware of the limited data available, however, we consider that an actualized panorama alerts on this gap of knowledge and helps to incentivize future research to bring data on human infections up to date in sites where animal infections have been recorded, and vice versa. Furthermore, our effort has now evidenced that it is important to consider Relapsing Fever as a differential diagnosis in cases currently diagnosed as “Fever of Unknown Origin” in Mexico.

***(2) Major Comments:
1. The detailed genomic description of the first documented endemic case of Bbss in Mexico is certainly original and should be the major focus of the report. The manuscript should include the actual BLAST sequence from that case to confirm its validity. Starting with this case will show where future studies should go in determining the epidemiology of tick-borne disease in Mexico. In addition, a detailed travel history should be given because the patient may have acquired his infection outside of Mexico.***

**Response**: We now include travel information, as detailed as the patient reported it (Lines 119-123). We were careful to assess whether he had made trips outside of Mexico, yet he only reported local trips within the state of Veracruz. We agree that this case sheds some light as to where future epidemiological studies should go and which can be enriched by the most complete information to date on the current epidemiological panorama of Borreliosis in Mexico, that we now present.

We now include the BLAST sequence in the manuscript to confirm the validity of the case (Lines 168-173

***(3) The epidemiology of Borrelia burgdorferi (Bb) and relapsing fever Borrelia (RFB) in Mexico is certainly of interest, but the available data was limited to 18 states, thereby introducing significant selection bias into the study results. Presentation of this review data could be done in a more organized and concise format with less speculation about the implications of the incomplete data.***

**Response:** We thank the reviewer and have now re-organized the manuscript into sections (A), related to the case report and section (B) related to review of the literature. The case report is now separated and fully presented after the introduction (Lines 114-180).

The epidemiological panorama is more organized, and less speculation is made about the incomplete epidemiological panorama of borreliosis in Mexico. We also discuss this point (Lines 374-379, 380-383, 446)

***(4) A benefit of separating out the case report from the epidemiology is that the case can be presented in a more organized fashion, with clinical features, test methodology, results and conclusions. Right now the clinical and epidemiology aspects are mixed together, and it is a mess.***

**Response:** After the reviewer's assertive comment of placing the case report as the center of attention, we consider that the case report and the epidemiological panorama of borreliosis are now more understandable.

***(5) Page 4, line 84: "The competent vectors of this bacterial species are hard ticks of the Ixodes ricinus complex". Ixodes ricinus is mainly found in Europe, so better to say that Bb is transmitted by hard ixodid ticks. The authors should also mention soft argasid ticks that transmit RFB (Fesler et al, Healthcare 2020;8:97).***

**Response:** We made the pertinent change about the *Ixodes ricinus* complex (Lines 94-95). Concerning to RF vectors, the information about that soft ticks are the main vectors are present in Lines 85-87.

***(6) For future review, it would be helpful to separate the Tables from the text. Much easier to follow the bouncing ball that way.***

**Response:** We agree, however, the format of PLoS ONE indicates that tables must be placed next to the paragraph where they were referred for the first time.

***(7) Page 15, line 221: Isn't it surprising that Bb was reported mostly in urban communities? The number of recognized RFB cases (5) was too small to draw geographical conclusions.***

**Response:** We agree that is surprising that LD cases were reported mostly in urban communities. On the other hand, as the reviewer says, the number of RF cases is too small, for this reason, we discuss the most likely reason of this situation (Lines 380-383).

***(8) Numerous spelling and grammatical errors need to be corrected. Examples (comments): prompts to include both diseases as possible causes of reports on “fever of unknow origin” (awkward sentence and sp), disperse (sp), incriminated (implicated), widely dispersed (just the opposite: not disseminated), a red skin lesion after visiting the family farm that lasted several weeks (the lesion, not the visit), sensibility (sensitivity), short time (short term), starting performing (?), BL(LB?), sensorial (sensory), sub-sampling (inadequate sampling).***

**Response:** We made the pertinent grammatical and spelling changes as the reviewer suggested (Lines 51, 85, 87, 105, 126, 135, 369, 373, 401, 422, 446, 492-493).

***Minor Comments:
(9) Page 22, line 331: REP and MAB sentence is hard to understand***

**Response:** We eliminated the clarification in parenthesis, to make it easier to read (Line 369).

***(10) Reference 41 is incomplete***

**Response:** We apologize and completed the reference 67 (Lines 694-696).

75.	Guzmán-Cornejo C, Robbins RG. The genus Ixodes (Acari: Ixodidae) in Mexico: adult identification keys, diagnoses, hosts, and distribution. Rev Mex Biodivers. 2010;80: 289–298.

**REVIEWER #4**

***(1) The author could improve the manuscript by citing references from the actual work that reported the Borrelia species, rather than utilising single review article (Reference 3 and 10), that does not refer to the work done on the pathogen by initial authors. This is especially relevant to the tables.***

**Response:** We thank the reviewer and now placed the references of the original descriptions for each species (Table 1).

***(2) Abstract
Line 31
Insert full stop after human and start a new sentence with Women: species were reported in a total of 1,347 reports, of which 398 were of humans. Women and children from rural communities appear to be…***

**Response:** We made the suggested changes (Line 34).

***(3) Line 36, only made from 18 states out of how many states?***

**Response:** We specified the total number of states (Line 39).

***(4) Introduction
Table 1. Pathogenic Borrelia species:
The first group on table 1 is RF not FR.***

**Response:** We apologize and corrected the error on Table 1.

***(5) On the Relapsing fever transmitted by ticks in Transcaucasia
The proper references for Candidatus Borrelia kalaharica should be:
1. Fingerle V, Pritsch et al. 2016. “Candidatus Borrelia kalaharica” detected from a febrile traveller returning to Germany from vacation in southern Africa. PLoS Negl Trop Dis 10:e0004559. <https://doi.org/10.1371/journal.pntd.0004559>.
2. Cutler S.J., Ahmed A.O., et al, 2018. Ornithodoros savingyi – the tick vector of Candidatus Borrelia kalaharica in Nigeria. Journal of Clinical Microbiology. 56(9): e00532-18.*** [***https://jcm.asm.org/content/jcm/56/9/e00532-18.full.pdf***](https://jcm.asm.org/content/jcm/56/9/e00532-18.full.pdf)

**Response:** We thank the reviewer and now include both references (Table 1).

***(6) Methods
Line 104: include year of coverage of studies 1939 and 2020***

**Response:** We made the pertinent changes (Lines 184-185).

***(7) Line103-110 refers to the methodology used for literature search for mammals, human cases and ticks in Mexico. What is line117-123 meant to infer? Are they additional database for human studies?***

**Response:** Since notification of borreliosis is not mandatory in Mexico, some published case reports and serological surveys of the human Mexican population has been published without being included in the official databases. The opposite also holds true, where some hospitals and clinics voluntarily notify cases of Lyme disease, yet this information is not necessarily published in scientific journals and therefore do not include symptoms nor detailed information. For this reason, we only included them in the geographic analyses. To make clarify this situation, we included this statement in the manuscript (Lines 197-200, 204-205).

***(8) Line 149: DNA was extracted from? Blood or Sera? Please clarify and include in the sentence***

**Response:** We made the clarification (Lines 140).

***(9) Results
Table 2: Include a first column for LB and RF groups:
Group Borrelia species No. of records Type of host
LB***

**Response:** We accept and made the pertinent changes (Table 2).

***(10) Discussion
Line 305: Brumpt what year?
Line 308: Pilz and Mooser what year?
Line 315: Salinas-Meléndez et al.include the year***

**Response**: We followed the instructions of the journal reference style that only cites the author(s), without including the year.

***(11) Line 336: Did you mean BL or LB*?**

**Response**: We apologize for the error. The correct abbreviation must be LB, which is now changed (Line 373).

***(12) The discussion is realistic with findings.***

**Response**: We really appreciate your comment

**REVIEWER #5**

***There are very few points which might need clarification.***

***(1) In the table 1 the authors have gathered information using one manual of systematics and some case studies and articles. Is the list trying to be exhaustive? At least I know some species, e.g. Borrelia finlandensis, which according to some references should be a species of its own and it is not mentioned in the table. Thus, a wider description of how species were chosen and if the list is covering all pathogenic species might be useful addition. Also in the introduction it might be good to tell the readers about latest development in Borrelia -systematics, e.g, division of the genus into Borrelia and Borreliella. Also term Borreliella might be useful to add as keyword in database search.***

**Response**: In Table 1 we did not intend to report an exhaustive search on all *Borrelia* species of the literature. The intention was to only include *Borrelia* species that are pathogenic for humans and/or animals, and therefore many species are not listed. We specified this in the text (Lines 87-95) stating that 25 of the RF group and 11 of the LB group were capable of causing diseases in human or animal populations. To avoid confusion, we change and referred to the original description articles, where the pathogenic species were firstly described, according to Reviewer 4.

We appreciate the recommendation to inform to the reader on the latest taxonomic updates of this genus. We therefore included a paragraph in the introduction regarding this point (Lines 77-84). Additionally, we followed the recommendation and included “Borreliella” as a keyword.

***(2) As not all readers are familiar with zoology, it might be aid readability and be educational if existing common names of animal species could be added to the table 3.***

**Response:** We are grateful for your recommendation. We now included English and Spanish common names in Table 3.

***(3) In the map figures the figure legends and numbers in the actual figures are not very informative. It might be not clear to reader what is meant “records of borrelia” for example in the figure 2. Does it mean “amount of borrelia findings from humans and animals isolated during 20xx-20xx” or something else? The figure legends should be carefully checked and more informative.***

**Response**: We reviewed all Figure legends, and modified those of Figures 2 and 3.

***(4) In the beginning of discussion it would be interesting to know, when the first borrelia findings were made.***

**Response**: The first record of *Borrelia* in Mexico, as well as the first human case and animal exposure are detailed in the first paragraph of the Discussion. Possibly the misunderstanding arose because we erroneously stated “case” instead if “record”, which was now modified (Line 342).

***(5) Minor points:
- Instead of “sex” maybe gender could be used (page 15, line 213)***

**Response**: We replaced “sex” by “gender” (Line 249).
