# Supplementary material for: Lyme disease and relapsing fever in Mexico: An overview of human and wildlife infections
Source: PLoS One. 2020 Sep 17;15(9):e0238496. doi: 10.1371/journal.pone.0238496 (PMC7497999; doi:10.1371/journal.pone.0238496)
Supplement: Attachment — Submitted filename: Response to Reviewers.docx [file pone.0238496.s005.docx]

**Response to Reviewers**

We thank reviewer #3 for the comments and have addressed all the points making the pertinent changes throughout the manuscript. The detailed responses are included below.

**REVIEWER #3**

***General comment***

***The authors have done an excellent job of revising the manuscript as suggested by the reviewers. Several remaining spelling and grammatical errors need to be corrected.***

**Response**: We are very thankful with your comment.

***1. Lines 28-30: "The aims of this study were (1) to present the first confirmatory evidence of an endemic case of Lyme disease in Mexico and (2) to analyze the epidemiological trend of these xxx diseases...."***

**Response**: We made the pertinent changes (Lines 28-29).

***2. Lines 35, 61: "...were shown to be..."***

**Response**: We accept and made the changes (Lines 35, 61).

***3. Line 40: "...of performing..."***

**Response**: We made the pertinent change (Line 40).

***4. Lines 42-43: "Finally, the search for Borrelia...is recommended..."***

**Response**: We modify the sentence as proposed by the reviewer (Line 44).

***5. Line 115: "in August 2017"***

**Response**: We made the pertinent change (Line 117).

***6. Line 416: "apyrexia"***

**Response**: We accept and made the change (Line 415).

***7. Line 418: "exanthems"***

**Response**: We corrected the spelling (Line 417).

***8. Line 422: "The analysis of Borrelia...has shown..."***

**Response**: We made the pertinent change (Line 421).

***9. Line 437: "... which affect several host species (rodents, deer and dogs)."***

**Response**: We accepted and made the pertinent changes (Line 436).

***10. The Case Report heading on Line 114 should have a subheading of "Clinical Summary and Test Procedures" to match the "Results" subheading on Line 157. Line 132 should be a new paragraph.***

**Response**: We agree with the suggestions of the reviewer and made the pertinent changes (Lines 115, 135).

***11. In addition, the authors should add one recent reference that supports their observations:***

***Fesler MC, Shah JS, Middelveen MJ, Burrascano JJ, Stricker RB. Lyme disease: diversity of Borrelia species in California and Mexico based on a novel immunoblot assay. Healthcare (Basel) 2020; 8:97.***

**Response**: We were aware of this publication. However, we had decided not to include it because the study was not done in Mexico. We only included studies done in Mexico, as was established in the methods (Lines 184-186). Additionally, since the study did not report the place from where the Mexican patients had come, they could not be included in the geographical analysis of our work. Both these points were the reasons for not having considered this reference in our current study.
